# Pontocerebellar hypoplasia type 9 with a novel combination of compound heterozygous variants in *AMPD2*

**DOI:** 10.1038/s41439-026-00348-0

**Published:** 2026-04-23

**Authors:** Shuhei Dohi, Junko Hotta, Kosuke Ito, Tomoyo Yamashita, Chie Ono, Satoru Sakuma, Kazuyuki Komatsu, Ken Inoue, Hirotomo Saitsu, Takashi Hamazaki, Toshiyuki Seto

**Affiliations:** 1 Department of Pediatrics, Osaka Metropolitan University Graduate School of Medicine, Osaka, Japan; 2 Department of Medical Genetics, Osaka Metropolitan University Graduate School of Medicine, Osaka, Japan; 3 Department of Biochemistry, Hamamatsu University School of Medicine, Hamamatsu, Japan; 4 Medical Genome Center, National Center of Neurology and Psychiatry, Tokyo, Japan

**Keywords:** Neurodegeneration, Clinical genetics

Pontocerebellar hypoplasia type 9 (PCH9; OMIM#615809) is a rare autosomal recessive neurodevelopmental disorder caused by biallelic variants in the *AMPD2*, which encodes adenosine monophosphate deaminase 2. PCH9 is characterized by progressive microcephaly, severe developmental delay, spasticity, hypomyelination, pontocerebellar hypoplasia and a characteristic ‘figure-of-8’ appearance of the midbrain on magnetic resonance imaging (MRI)^1^. Here, we report a 3-year-6-month-old Japanese girl with severe developmental delay and microcephaly.

She was born at term after an uncomplicated pregnancy and delivery: weight 3,120 g (+0.33 standard deviation (s.d.)), length 48.0 cm (−0.54 s.d.), head circumference 32.6 cm (−0.50 s.d.). Her motor development was profoundly delayed, with absence of head control and visual tracking. She did not babble or smile, had nystagmus without visual fixation, and exhibited marked spasticity with hyperreflexia, joint contractures, pes equinus and truncal hyperextension. Clonus was absent, and cerebellar ataxia symptoms were not evident. At 3 years and 6 months of age, her growth parameters were as follows: weight 11.8 kg (−1.47 s.d.), height 84.1 cm (−3.30 s.d.) and head circumference 45.8 cm (−4.6 s.d.). Although she had not yet achieved head control, rehabilitation improved limb hypertonia. She was babbling and smiling. No epileptic seizures were observed at the time of evaluation, and she could breathe spontaneously and consume finely chopped food orally. Her parents were nonconsanguineous, and the family history was unremarkable, except for an older brother with bilateral sensorineural hearing loss due to compound heterozygous *GJB2* (NM_004004.6): c.[235delC];[134G>A;408C>A] (p.[Leu79fs];[Gly45Glu;Tyr136Ter]).

Extensive metabolic, biochemical, electrophysiological, infectious, ophthalmological and otolaryngological evaluations, including cerebrospinal fluid analysis, nerve conduction studies, polymerase chain reaction testing for cytomegalovirus using a preserved dried umbilical cord specimen^2^, electrocardiography, ultrasonography and auditory brainstem response testing, revealed no notable abnormalities. Chromosomal G-banding revealed a normal female karyotype (46, XX).

Brain MRI at 6 months of age revealed severe hypomyelination and hypoplasia of the brainstem, corpus callosum and cerebellum. Although myelination was markedly delayed, gradual progression was observed. MRI performed at 3 years and 6 months demonstrated hypoplasia of the corpus callosum; atrophy of the pons, cerebellum and basal ganglia; and a characteristic ‘figure-of-8’ appearance of the midbrain (Fig. 1).

**Fig. 1:**
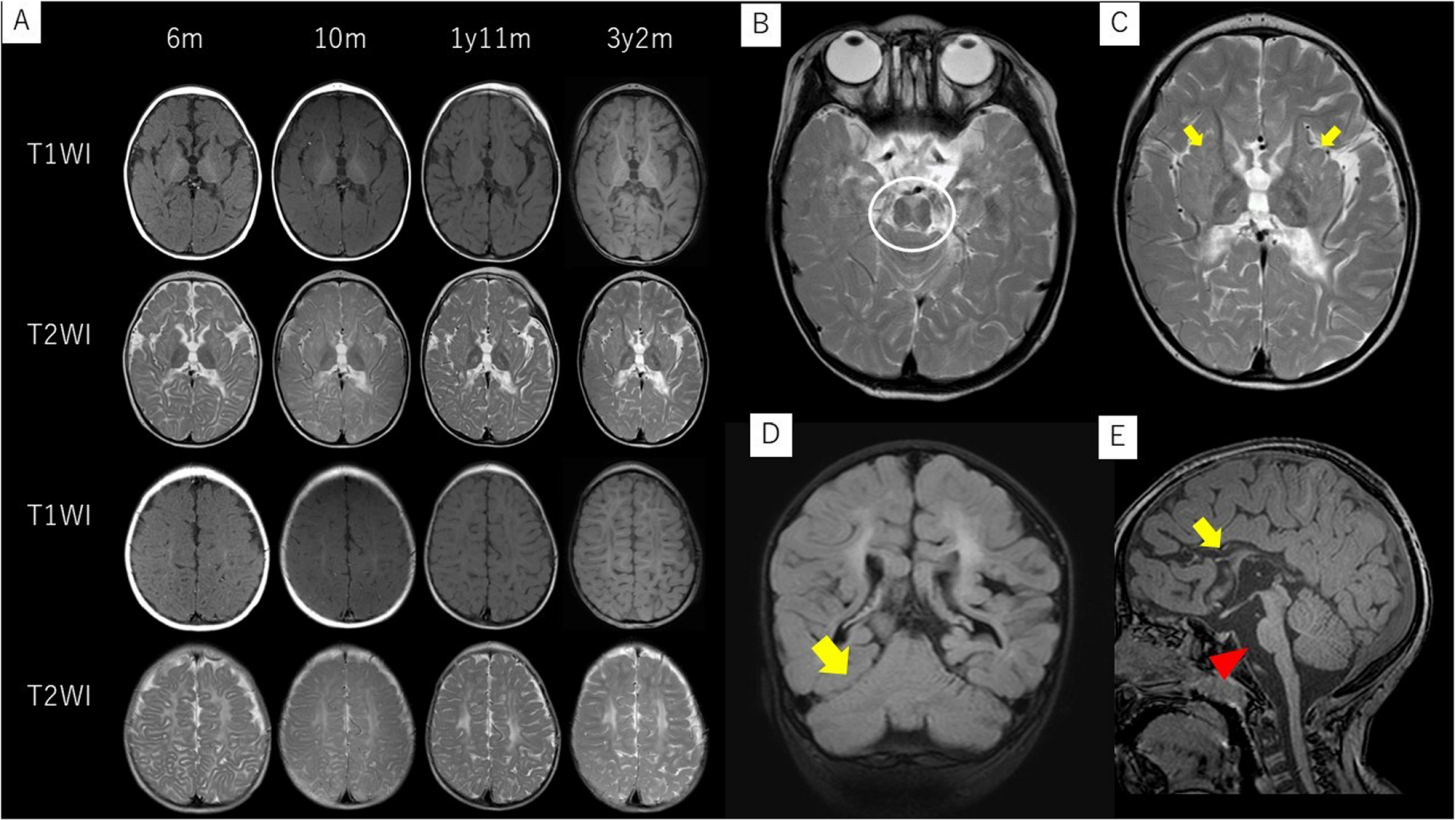
Brain MRIs from this case. **A** Axial T1-weighted and T2-weighted images are shown alternately. From left to right, images were obtained at 6 months, 10 months, 1 year and 11 months, and 3 years and 2 months of age. Myelination is severe delayed but shows slow progression over time. **B**, **C** Axial T2-weighted images show characteristic “figure of 8” midbrain appearance (white ovals) and small basal ganglia (yellow arrows). **D** Coronal T2-FLAIR images reveal hypoplasia/atrophy of the cerebellar hemispheres with relative sparing of the vermis (yellow arrows). **E** Sagittal T1-weighted images show small pons (red arrowheads) and extreme thinning of corpus callosum (yellow arrows). MRI magnetic resonance imaging.

Based on the combination of hypomyelination and basal ganglia involvement, leukodystrophy, including hypomyelination with atrophy of the basal ganglia and cerebellum, was initially suspected^3^. Targeted genetic analyses, including panel sequencing using the Agilent ClearSeq Inherited Disease Panel (3,204 genes) and focused testing for genes associated with congenital cerebral hypomyelination (including *PLP1*, *GJC2*, *TUBB4A*, *MBP*, *SLC16A2*, *HSPD1*, *SLC17A5*, *POLR3B*, *FAM126A*, *POLR3A* and *SOX10*), as well as an extended secondary analysis of 579 genes, including *UFM1*, identified no pathogenic variants.

Subsequently, whole-exome sequencing (WES) was performed for the proband. WES was outsourced to the Genetic Information Research Center, Research Institute for Microbial Diseases, Osaka University. Following capture with the Twist Exome 2.0 kit (Twist), libraries were sequenced on a NovaSeq 6000 Illumina platform with 101-bp paired-end reads. Variant calling was performed using HaplotypeCaller and DeepVariant. The protein-coding regions of MANE (Matched Annotation from NCBI and EMBL-EBI) genes were adequately covered, with 98.5% of these regions covered by at least 20 reads, ensuring sufficient analytical accuracy. The pathogenicity of variants was assessed according to the American College of Medical Genetics and Genomics (RRID: SCR_005769) guidelines^4^.

WES identified two heterozygous variants in *AMPD2* (NM_001368809.2) in the proband. These variants were confirmed by Sanger sequencing in trio samples: a maternally inherited frameshift variant, c.671_672del (p.(Leu224Argfs6)), and a paternally inherited frameshift variant, c.2444_2469del (p.(Glu815Alafs55)) (Fig. 2).

**Fig. 2:**
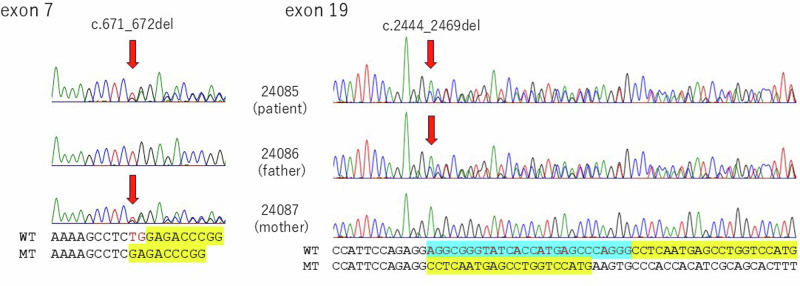
Sanger sequencing analysis of AMPD2 variants identified in the proband and her parents. Chromatograms show a frameshift deletion in exon 7 (NM_001368809.2:c.671_672del, p.(Leu224Argfs*6)) and a frameshift deletion in exon 19 (NM_001368809.2:c.2444_2469del, p.(Glu815Alafs*55)). The proband carries both variants in a compound heterozygous state. Segregation analysis demonstrated maternal inheritance of the c.671_672del variant and paternal inheritance of the c.2444_2469del variant. Arrows indicate the positions of the deletions. Corresponding wild-type (WT) and mutant (MT) nucleotide sequences are shown below each chromatogram.

The maternal variant (c.671_672del) was not registered in either the gnomAD v4.1.0^5^ or the 54KJPN^6^ database. This variant is located in exon 7, which is expressed in most *AMPD2* transcripts, and is predicted to result in mRNA degradation via nonsense-mediated decay. By contrast, the paternal variant (c.2444_2469del) was registered at a very low frequency in gnomAD v4.1.0 (allele frequency 6.90 × 10⁻⁶). This variant is located in the final exon and is predicted to cause a frameshift from glutamic acid at position 815, resulting in an aberrant C-terminal sequence of 55 amino acids beyond the normal stop codon.

According to the American College of Medical Genetics and Genomics criteria and ClinGen variant classification guidelines, the maternal variant, c.671_672del, was classified as likely pathogenic based on PVS1 and PM2_supporting. The paternal variant, c.2444_2469del, was classified as a variant of uncertain significance (VUS), supported by PVS1_Moderate, PM2_supporting and PM3, reflecting its location in the final exon, predicted escape from nonsense-mediated decay, and confirmation of a trans configuration with a likely pathogenic allele.

Pathogenic *AMPD2* variants cause two clinically distinct disorders: PCH9 and spastic paraplegia type 63^1^. The genetic analysis results and her clinical phenotype were discussed by a multidisciplinary genetics and pediatrics committee, which ultimately concluded that the compound heterozygous *AMPD2* variants were associated with PCH9.

Radiological features observed in this case included pontine hypoplasia or atrophy with cerebellar atrophy on coronal images, reduction in the size of the pons and middle cerebellar peduncles, an abnormal midbrain configuration with a characteristic ‘figure-of-8’ appearance on axial images, diffuse loss of cerebral white matter^7^, and absence or marked thinning of the corpus callosum. These phenotypes of MRI findings have been described as characteristic of PCH9 and are useful for differentiating it from other PCH subtypes^8,9^, further supporting the diagnostic certainty in the present case. Small basal ganglia and thalamic hypoplasia have also been reported^8^; therefore, careful observation must be continued. Recent studies have suggested a molecular mechanism for neurodegeneration caused by disruption of purine nucleotide metabolism due to *AMPD2* variants, as well as regional differences in vulnerability to neurodegeneration^10^. This may be related to differences in the severity and distribution of neuroimaging findings among cases. It is important to continue accumulating both genetic variants and detailed clinical phenotypes from patients with PCH9 whose genotypes have been identified. Interestingly, to our knowledge, no reports have demonstrated a relationship between the compound heterozygous *GJB2* variant observed in her brother and PCH9^11^.

In this case, the paternal *AMPD2* variant was evaluated as a VUS. Frameshift variants located in the final exon require careful interpretation because nonsense-mediated decay is not expected to occur, allowing the production of truncated or altered proteins. In such cases, pathogenicity depends on whether the affected C-terminal region is critical for protein function^12^. Although the present variant alters the C-terminal sequence of *AMPD2*, the full-length *AMPD2* protein consists of 825 amino acids^13^, and the predicted truncation affects fewer than 10% of the protein length. Furthermore, current domain annotations based on InterPro/Pfam indicate that the canonical AMP deaminase domain does not extend to this region^14^. Therefore, in the absence of functional evidence, a classification beyond moderate loss-of-function evidence was not justified. The application of phenotype-based criteria was limited by the genetic heterogeneity of pontocerebellar hypoplasia, and upgrading loss-of-function evidence was constrained by the absence of reported pathogenic variants downstream of this alteration and by the lack of functional data supporting the critical role of the C-terminal region^15^. However, these criteria may become applicable in the future as the clinical and genetic spectrum of PCH9 becomes more comprehensively delineated. For example, further refinement of diagnostic yield estimates specific to PCH9 and the accumulation of additional evidence clarifying the functional relevance of the C-terminal region of *AMPD2* may facilitate an upgrade of these classifications.

In summary, we report a case of a Japanese patient with clinical and neuroimaging features consistent with PCH9, in whom WES identified compound heterozygous *AMPD2* variants, including a likely pathogenic variant and a VUS. This case refines the genetic spectrum of *AMPD2* and highlights the importance of careful variant interpretation in rare neurodevelopmental disorders.

## HGV Database

The relevant data from this Data Report are hosted at the Human Genome Variation Database via Figshare at 10.6084/m9.figshare.hgv.3645 (ref. ^16^) and 10.6084/m9.figshare.hgv.3648 (ref. ^17^).
